# Exploring the factors related to adolescent health literacy, health-promoting lifestyle profile, and health status

**DOI:** 10.1186/s12889-021-12239-w

**Published:** 2021-12-01

**Authors:** Fen Chu-Ko, Meng-Ling Chong, Chi-Jung Chung, Chun-Chi Chang, Hsin-Yi Liu, Li-Chi Huang

**Affiliations:** 1 Nursing Department, Jenteh Junior College of Medicine, Nursing and Management, Miaoli County, Taiwan; 2 Department of Public Health, China Medical University, Taichung, Taiwan; 3 Department of Nursing, Tungs’ Taichung MetroHarbor Hospital, Taichung, Taiwan; 4 School of Nursing, China Medical University; Adjunct Supervisor, Department of Nursing, China Medical University Children Hospital, Taichung, Taiwan

**Keywords:** Adolescent, Health literacy, Health-promoting lifestyle profile, Health status, Exercise

## Abstract

**Background:**

Health literacy has been concerned a key factor for determining the use of health information and promoting health. The study aimed to explore adolescent health literacy, health-promoting lifestyle profile, and health status and related factors.

**Methods:**

A cross-sectional study design was used; 918 first year junior college students were recruited in Taiwan. The measurements were the Chinese Health Literacy Survey Questionnaire (HLS-C-Q), the Chinese Health-Promoting Lifestyle Profile (HPLP-S), and the Health Status Questionnaire.

**Results:**

The mean score for health literacy was 36.15 (±6.21), with 30.17% of the participants having insufficient or problematic health literacy. Further, 19.9% of participants were obese and 11.2% experienced emotional instability. Health literacy and health-promoting lifestyle profile showed significant positive and negative correlations with perceived health status and depression, respectively (*p* < 0.05). An exercise frequency of ≥3 times/week was a predictor of health literacy, health-promoting lifestyle profile, and emotional stability.

**Conclusions:**

Adolescent health literacy, health-promoting lifestyle profile, and health status require careful consideration. In adolescents, developing regular exercise may increase health literacy, thereby developing healthy lifestyle profiles and ameliorating obesity and depression-related issues.

**Supplementary Information:**

The online version contains supplementary material available at 10.1186/s12889-021-12239-w.

## Background

The concept of health literacy was first proposed by Simonds (1974) in *Health Education as Social Policy,* which emphasized the importance of health literacy on national health and the provision of the most basic health literacy education for students in schools [1]. Further, Baker (2006) proposed that health literacy is an important predictor of health status and outcomes [2]. Health literacy has been considered a key factor for determining the effective use of health information and making choices for promoting health [3]. Individuals with low health literacy have a limited understanding of health information and low health self-management ability, which increases hospitalization and medical expenses and causes high mortality [4]. Good health-promoting behavior and lifestyle habits are developed during adolescence, a vital developmental stage, which involves physical, psychological, and social changes that will affect the adolescents’ quality of life in adulthood [5]. Early health literacy can aid in individuals gaining an understanding of health information and promoting interaction with the healthcare system, thereby providing positive health outcomes in the future [6]. Therefore, understanding the health literacy of adolescents is crucial for personal health. The health behavior of an adolescent plays an important role in developing a healthy lifestyle, which may affect his/her lifelong health. However, several studies of health literacy have focused on elucidating the effects of poor health literacy on adult health and few studies have involved adolescents [4, 7, 8]. A study of adolescents using an online survey in Germany found 8.4% of participants had difficulty understanding the health information and 22.7% of adolescent had a low level of health knowledge [8]. However, in a systemic review of 17 studies on adolescent health literacy, the definitions of health literacy were inconsistent across studies because different conceptual frameworks and assessment tools were used [9]. Adolescence is the good opportunity period for learning and improving health literacy during development. Thus, an understanding of factors related to adolescents’ health literacy, health-promoting lifestyle profile, and health status is essential for promoting adolescent health.

### Health literacy

The World Health Organization (WHO) has proposed that health literacy represents the cognitive and social skills that determine the motivation and ability of individuals to access, understand, and use information for promoting and maintaining good health [10]. Sørensen et al. (2012) proposed a comprehensive definition of health literacy as entailing individuals’ knowledge, motivation, and competences to access, understand, appraise, and apply health information for making decisions in everyday life concerning healthcare, disease prevention, and health promotion for better quality of life [3]. Health literacy further affects healthcare and medical expenses, health behavior, and care effectiveness in individuals as well as public participation and empowerment and is associated with the issues of fairness and sustainability [11].

In 2011–2012, the European Consortium Health Literacy Project employed the European Health Literacy Survey Questionnaire (HLS-EU-Q) to conduct a large-scale health literacy survey in eight European countries. The survey results reported that 12% of participants had insufficient health literacy and 47% had limited (insufficient or problematic) health literacy [12]. Duong et al. (2017) conducted a health literacy survey with 10,024 general public participants and found that the mean health literacy of the people in each country was: Indonesia 31.4; Kazakhstan 31.6; Malaysia 32.9; Myanmar 31.3; Taiwan 34.4; and Vietnam 29.6, show that, with the exception of Taiwan, most people in these countries lacked health literacy based on a problematic health literacy score of ≤ 34 [13]. Hence, health literacy was found to be typically insufficient in the general public in different countries. However, there is a lack of research data on health literacy in adolescents.

Studies found that age, gender, education level, family income, and occupation were the demographic variables correlated with adults’ health literacy [12, 14]. Loer et al. (2020) also found that age, gender, education, social support and self-efficacy were associated with adolescents’ health literacy [8]. In addition, health literacy is associated with smoking, alcohol consumption, exercise, and health behavior in the adult population [10, 12, 13]. Studies found that higher health literacy is associated with higher ability to pay for medication, higher self-perceived social status, and higher community involvement in adults [12–16]. Lee, Lee, and Moon (2016) found that health literacy can directly affect self-care activities in diabetics [17]. People with low health literacy have poorer health and lack self-related health knowledge and self-care ability.

### Health-promoting lifestyle profile

The WHO organized the first international conference on health promotion in 1986 [18]. In the resulting Ottawa Charter, a definition was proposed for health promotion: “Health promotion is the process of enabling people to increase control over their health and its determinants and thereby improve their health”. Walker, Sechrist, and Pender (1987) defined health-promoting lifestyle profile as a multi-dimensional pattern of self-initiated actions and perceptions that serve to maintain or enhance the level of wellness, self-actualization, and fulfillment of the individual [19]. These dimensions include self-actualization, health responsibility, nutrition, interpersonal support, stress management, and exercise, which is a framework used in several subsequent studies [20].

Factors related to health-promoting lifestyle profile include gender and age [20]. Married individuals have higher health-promoting lifestyle profile than single individuals. With age, the health responsibility score increases, whereas the stress management score decreases [21]. The health-promoting lifestyle profile of university students significantly differs according to their academic major, education level, age, and financial status, and this profile has an important correlation with health status [22]. An individual’s lifestyle profile is developed during whole lifespan. Thus, effective health promotion and disease prevention strategies are crucial for development during adolescence.

### Adolescent health

The lifestyle profile of adolescents is inevitably affected by emerging technology and obsession with electronic equipment has become threat to their health and growth. An unhealthy lifestyle characterized by the lack of physical exercise is widespread in adolescents. Bhatti et al. (2020) surveyed adolescents aged 16–18 years and found that 15% of adolescents smoked, 21% were overweight or obese, 80% did not achieve the daily required intake of vegetables and fruits, and 90% did not take enough daily exercise in the UK [23]. Moreover, 55% of adolescents do not have sufficient daily sleep or experience insomnia, 52% do not have an exercise habit, and 3% experience anxiety or depression in Taiwan [24]. Nevertheless, surveys have shown that the amount of physical activity is less in adolescents and most adolescents’ age of 15 years did not follow the WHO recommendations of ≥60 min of moderate-to-vigorous activity daily [25].

Adolescence is a period of intense changes during life expansion, with drastic changes in both physiological and psychological growth, which affects an individual’s lifestyle profile and health behavior, thereby affecting health in adulthood [26]. Therefore, elucidating health literacy of adolescents is crucial for both a healthy lifestyle profile and health. The aim of this study was to explore adolescent health literacy, health-promoting lifestyle profile, health status and related factors. Specifically, the research question guiding the study was: ‘What are the demographic factors related to health literacy, health-promoting lifestyle profile, and health status in adolescents in Taiwan?’

## Methods

### Participants and setting

A cross-sectional study design was employed. Participants, who were freshmen in junior college in September 2019 in central Taiwan, were recruited by purposive sampling. Participants’ inclusion criteria were first year junior college students. The sample size was estimated by using multiple regression. G-power calculations, type I error was set as α = 0.05, power was 0.95, the number of independent variables was 10, effect size was 0.03 (small), and estimated sample size was 823. Considering attrition, a 20% excess of participants was added and a total of 988 questionnaires were distributed; 968 were returned (97.98%) and 50 invalid questionnaires were excluded. The completion rate was 92.9% with 918 valid questionnaires remained. This high response rate could be because the survey was distributed to freshmen at the enrollment stage of school.

### Study instruments

The questionnaire comprised four parts: demographic data; health literacy; health promotion lifestyle profile; and health status. General information included academic major, gender, age, place of residence, ethnicity, family income, medical history, smoking history, alcohol consumption history, and exercise history.

#### Health literacy scale

The HLS-EU-Q developed by Sørensen et al. (2012) was used to evaluate the ability to obtain, understand, analyze, and apply information related to practice health related issues [3]. The instrument was translated and had valuable validity and reliability [13]. The questionnaire comprised 47 questions, and 4-point Likert scale was constructed, with 4 indicating easy and 1 indicating difficult. A total score of 0–25, ≧26–33, ≧34–42, and ≧43–50 points represented insufficient, problematic, sufficient, and excellent health literacy, respectively. Duong et al. (2017) conducted a survey in six Asian countries and demonstrated that the HLS-EU-Q47 has good construct validity, high internal consistency (Cronbach’s alpha > 0.90), with good convergent validity and absence of ceiling and floor effects [13].

#### Health-promoting lifestyle profile scale

This scale measures the six dimensions of health-promoting lifestyle profile, self-actualization, health responsibility, nutrition, interpersonal support, stress management, and exercise, which proposed by Walker, Sechrist, and Pender (1987) [19]. The Chinese simplified health-promoting lifestyle profile scale (HPLP-S) was used in this study [27]. There were four questions for each dimension, resulting in 24 questions. A four-point Likert scalewas used, with 4 being “always” to 1 being “never”. The internal consistency of e HPLP-S was Cronbach’s α 0.69 to 0.73 for the subscales and 0.90 for the entire scale; the convergent validity of subscales was good [27].

### Health status measurement

This scale contained physical data, self-reported health status and psychological health scale, which represented the health status of students. The self-reported health status represents the perceived health status and the score ranged from excellent (5) to extremely poor (1). The physical data included body mass index (BMI) and body fat percentage. The BMI was calculated using the formula of weight divided by height^2^, and body fat was measured using a body fat meter. Tung’s Depression Inventory for college students was used to measure psychological health. Items related to depression syndrome and sign such as: ‘I feel sad’; ‘No one cares about me’; ‘My chest is tight and stuffy’ were included. This scale contained 32 questions and scores range “never or extremely rare – less than one day” (0), to “often or always – five to seven days” (3) weekly. A total score of ≤28, 29–35, 36–51, and ≥ 52 represent stable emotions, floating emotions, heavy mood, and depressive emotions, respectively. These concepts are dichotomized into two dimensions, stable emotions (≤ 28) and unstable emotions (≥ 29). The internal consistency reliability of the scale was 0.95 and test-retest reliability was 0.84 [28].

### Statistical analyses

After questionnaires were collected, compiled, and decoded, SPSS Windows 25 was used for statistical analyses. A significance level of α = .05 was used. The frequency, percentage, mean, and standard deviation were used to present descriptive statistics for demographics, health literacy, health-promoting lifestyle profile, and health status data. Pearson’s correlation was used to test the correlation between variables. An independent t-test was used to compare the means of two groups while one-way ANOVA was used for multiple group comparisons. Multiple linear regression was used to test factors benefiting the outcome measurements and logistical regression was use for those outcomes where data were categorical.

## Results

### Participants’ characteristics

Most participants were majoring in nursing (48.91%, *n* = 49) and most were female 79.08% (*n* = 726). The mean age was 15.49 ± 0.52 years, range 15–17 years. Most students (56.64%, *n* = 520) lived in a dormitory. Most participants’ families were of a moderate financial status (70.48%, *n* = 647). Furthermore, 2.72% (*n* = 25) of participants experienced chronic disease, 1.96% (*n* = 18) were smokers, and only 19.28% (*n* = 177) exercised twice a week (Table 1).

**Table 1:** Demographic characteristics (*n* = 918)

Variable	N (%)	Variable	N (%)
**Gender**		**BMI (kg/cm**^**2**^**)**^**b1**^	
Female	726(79.08)	Underweight	
Male	192(20.92)	Female	58 (6.32)
**Age (Mean** ± SD)	15.49(±0.52)	Male	12 (1.31)
**Place of residence**	520 (56.64)	Normal	
Dormitory or rented accommodation		Female	451 (49.13)
Home	398 (43.36)	Male	100 (10.89)
**Ethnicity**		Overweight	
Hokkien	645(70.26)	Female	92 (10.02)
Hakka	204(22.22)	Male	22 (2.39)
Province	31(3.38)	Obese	
Aboriginal	38(4.14)	Female	125 (13.62)
**Family income**		Male	58 (6.32)
Above Well off (Rich Well off)	217 (23.64)	**Body fat (%)**^**b2**^	
Fair	647 (70.48)	Low	
Poor	54 (5.88)	female	38 (4.14)
**Medical history**		male	13 (1.42)
No	893(97.28)	Normal	
Yes	25(2.72)	female	202 (22.00)
**Smoking history**		male	40 (4.36)
No	900(98.04)	High	
Yes	18(1.96)	female	217 (23.64)
**Alcohol consumption history**		male	57 (6.21)
No	899(97.93)	Obesity	
Yes	19(2.07)	Female	269 (29.30)
**Exercise history**		Male	82 (8.93)
No	329 (35.84)	**Depression Inventory**	
Yes	589 (64.16)	stable emotions (≤28 points)	815 (88.78)
**Exercise Frequency (per week)**		unstable emotions (≥29 points)	103 (11.22)
0	329 (35.84)	**Health status**	
1 ~ 2 times	412 (44.88)	Very good	86 (9.37)
≥3 times	177 (19.28)	Good	213 (23.20)
**Health Literacy**^**b3**^		Fair	442 (48.15)
Insufficient	19 (2.07)	Poor	124 (13.51)
problematic	258 (28.11)	Very poor	53 (5.77)
sufficient	466 (50.76)		
excellent	175 (36.15)		

*BMI* body mass index

^b1^ BMI underweight (female:< 16.7)(male:< 16.9)

normal (female:16.7≦BMI < 22.7)(male:16.9≦BMI < 22.9)

overweight (female:22.7≦BMI < 25.2)(male:22.9≦BMI < 25.2)

obese (female:≥25.2)(male:≥25.2)

^b2^ Body fat Low (female:< 17%)(male:< 14%)

Normal (female:17%≦ Body fat < 24%)(male:14%≦ Body fat < 20%)

High (female:24%≦ Body fat < 30%)(male:20%≦ Body fat < 25%)

Obesity (female:≥30%)(male:≥25%)

^b3^Health Literacy total score of 0–25: Insufficient, ≧26–33: problematic, ≧34–42: sufficient, ≧43–50: excellent

### Health literacy

The mean score of health literacy of 918 participants was 36.15 (±6.21), of which 2.07% (*n* = 19), 28.11% (*n* = 258), 50.76% (*n* = 466), and 19.06% (*n* = 175) had insufficient, problematic, sufficient, and excellent health literacy, respectively. Therefore, participants with insufficient and problematic health literacy accounted for approximately 30% of participants (Table 1). The mean score for understanding health information was the highest, whereas that for appraising health information was the lowest (Table 2).

**Table 2:** Health Literacy Scale

Questions	Very difficult	Fairly difficult	Fairly easy	Very easy	Mean(±SD)	Sequence
	n (%)					
**Access information**					3.18(±0.39)	
1. find information about symptoms of illnesses that concern you?	1(0.11)	54(5.88)	628(68.41)	235(25.6)	3.19(±0.53)	25
2. find information on treatments of illnesses that concern you?	0(0)	82(8.93)	637(69.39)	199(21.68)	3.13(±0.54)	32
3. find out what to do in case of a medical emergency?	3(0.33)	166(18.08)	588(64.05)	161(17.54)	2.99(±0.61)	41
4. find out where to get professional help when you are ill?	0(0)	42(4.58)	501(54.58)	375(40.85)	3.36(±0.57)	5
17. find information about how to manage unhealthy behaviour such as smoking, low physical activity and drinking too much?	3(0.33)	41(4.47)	493(53.7)	381(41.5)	3.36(±0.58)	4
18. find information on how to manage mental health problems like stress or depression?	7(0.76)	99(10.78)	551(60.02)	261(28.43)	3.16(±0.63)	29
19. find information about vaccinations and health screenings that you should have?	2(0.22)	89(9.69)	580(63.18)	247(26.91)	3.17(±0.59)	27
20. find information on how to prevent or manage conditions like being overweight, high blood pressure or high cholesterol?	2(0.22)	76(8.28)	566(61.66)	274(29.85)	3.21(±0.59)	17
32. find information on healthy activities such as exercise, healthy food and nutrition?	3(0.33)	41(4.47)	568(61.87)	306(33.33)	3.28(±0.56)	10
33. find out about activities that are good for your mental well-being?	4(0.44)	71(7.73)	560(61)	283(30.83)	3.22(±0.6)	15
34. find information on how your neighborhood could be more health-friendly?	5(0.54)	65(7.08)	570(62.09)	278(30.28)	3.22(±0.59)	16
35. find out about political changes that may affect health?	17(1.85)	203(22.11)	511(55.66)	187(20.37)	2.95(±0.7)	45
36. find out about efforts to promote your health at work?	1(0.11)	99(10.78)	587(63.94)	231(25.16)	3.14(±0.59)	30
**Understand information**					3.19(±0.4)	
5. understand what your doctor says to you?	2(0.22)	67(7.3)	602(65.58)	247(26.91)	3.19(±0.56)	24
6. understand the leaflets that come with your medicine?	5(0.54)	122(13.29)	557(60.68)	234(25.49)	3.11(±0.63)	34
7. understand what to do in a medical emergency?	7(0.76)	205(22.33)	562(61.22)	144(15.69)	2.92(±0.64)	47
8. understand your doctor’s or pharmacist’s instruction on how to take a prescribed medicine?	3(0.33)	60(6.54)	574(62.53)	281(30.61)	3.23(±0.57)	12
21. understand health warnings about behaviour such as smoking, low physical activity and drinking too much?	0(0)	31(3.38)	492(53.59)	395(43.03)	3.4(±0.55)	2
22. understand why you need vaccinations?	6(0.65)	78(8.5)	538(58.61)	296(32.24)	3.22(±0.62)	14
23. understand why you need health screenings?	1(0.11)	33(3.59)	514(55.99)	370(40.31)	3.36(±0.56)	6
37. understand advice on health from family members or friends?	1(0.11)	47(5.12)	609(66.34)	261(28.43)	3.23(±0.54)	13
38. understand information on food packaging?	5(0.54)	132(14.38)	582(63.4)	199(21.68)	3.06(±0.62)	36
39. understand information in the media on how to get healthier?	4(0.44)	89(9.69)	602(65.58)	223(24.29)	3.14(±0.58)	31
40. understand information on how to keep your mind healthy?	6(0.65)	77(8.39)	568(61.87)	267(29.08)	3.19(±0.6)	23
**Appraise information**					3.14(±0.41)	
9. judge how information from your doctor applies to you?	1(0.11)	42(4.58)	506(55.12)	369(40.2)	3.35(±0.57)	7
10. judge the advantages and disadvantages of different treatment options?	6(0.65)	170(18.52)	562(61.22)	180(19.61)	3(±0.64)	39
11. judge when you may need to get a second opinion from another doctor?	5(0.54)	120(13.07)	609(66.34)	184(20.04)	3.06(±0.59)	37
12. judge if the information about illness in the media is reliable?	8(0.87)	173(18.85)	571(62.2)	166(18.08)	2.97(±0.64)	43
24. judge how reliable health warnings are, such as smoking, low physical activity and drinking too much?	0(0)	23(2.51)	482(52.51)	413(44.99)	3.42(±0.54)	1
25. judge when you need to go to a doctor for a check-up?	12(1.31)	187(20.37)	546(59.48)	173(18.85)	2.96(±0.67)	44
26. judge which vaccinations you may need?	17(1.85)	205(22.33)	522(56.86)	174(18.95)	2.93(±0.69)	46
27. judge which health screenings you should have?	13(1.42)	113(12.31)	553(60.24)	239(26.03)	3.11(±0.65)	33
28. judge if the information on health risks in the media is reliable?	5(0.54)	87(9.48)	565(61.55)	261(28.43)	3.18(±0.61)	26
41. judge where your life affects your health and wellbeing?	2(0.22)	67(7.3)	590(64.27)	259(28.21)	3.2(±0.57)	20
42. judge how your housing conditions help you to stay healthy?	2(0.22)	78(8.5)	576(62.75)	262(28.54)	3.2(±0.58)	19
43. judge which everyday behaviour is related to your health?	1(0.11)	48(5.23)	568(61.87)	301(32.79)	3.27(±0.56)	11
**Apply information**					3.17(±0.4)	
13. use information the doctor gives you to make decisions about your illness?	3(0.33)	51(5.56)	534(58.17)	330(35.95)	3.3(±0.58)	9
14. follow the instructions on medication?	1(0.11)	44(4.79)	475(51.74)	398(43.36)	3.38(±0.58)	3
15. call an ambulance in an emergency?	3(0.33)	66(7.19)	490(53.38)	359(39.11)	3.31(±0.62)	8
16. follow instructions from your doctor or pharmacist?	8(0.87)	112(12.2)	525(57.19)	273(29.74)	3.16(±0.66)	28
29. decide if you should have a flu vaccination?	8(0.87)	92(10.02)	523(56.97)	295(32.14)	3.2(±0.64)	18
30. decide how you can protect yourself from illness based on advice from family and friends?	5(0.54)	54(5.88)	610(66.45)	249(27.12)	3.2(±0.56)	21
31. decide how you can protect yourself from illness based on information in the media?	14(1.53)	148(16.12)	586(63.83)	170(18.52)	2.99(±0.64)	40
44. make decisions to improve your health?	6(0.65)	84(9.15)	555(60.46)	273(29.74)	3.19(±0.62)	22
45. join a sports club or exercise class if you want to?	17(1.85)	139(15.14)	532(57.95)	230(25.05)	3.06(±0.69)	35
46. influence your living conditions that affect your health and wellbeing?	14(1.53)	145(15.8)	552(60.13)	207(22.55)	3.04(±0.67)	38
47. take part in activities that improve health and well-being in your community?	18(1.96)	172(18.74)	544(59.26)	184(20.04)	2.97(±0.68)	42
**Total score**					36.15 (±6.21)	

### Health-promoting lifestyle profile (HPLP)

The mean score for health-promoting lifestyle profile was 60.76 (±11.88), which was considered moderate. A standard deviation above and below the mean score was used as cutoff points [25]. Accordingly, 7.41% (*n* = 68), 84.97% (*n* = 780), and 7.63% (*n* = 70) of participants were in the high, moderate, and low health-promoting lifestyle profile groups, respectively. Among the six dimensions of HPLP, the interpersonal support was the highest, whereas that for health responsibility was the lowest (Table 3).

**Table 3:** Health-promoting lifestyle profile scale

Items	Never	Sometimes	Often	Always	Mean(±SD)	Sequence
	n (%)					
**Exercise**					2.28(±0.57)	
1. Perform stretching exercises at least 3 times per week.	89(9.69)	571(62.2)	182(19.83)	76(8.28)	2.27(±0.75)	18
6. Participate in supervised exercise programs or activities.	109(11.87)	433(47.17)	255(27.78)	121(13.18)	2.42(±0.86)	16
10. Check my pulse rate when exercising.	403(43.9)	352(38.34)	116(12.64)	47(5.12)	1.79(±0.85)	24
16. Engaga in recreational physical activities.	47(5.12)	410(44.66)	297(32.35)	164(17.86)	2.63(±0.83)	12
**Nutrition**					2.5(±0.54)	
2. Choose foods without preservatives or other additives.	108(11.76)	585(63.73)	188(20.48)	37(4.03)	2.17(±0.68)	21
3. Eat 3 regular meals a day.	40(4.36)	296(32.24)	334(36.38)	248(27.02)	2.86(±0.87)	6
8. Include roughage/fiber (whole grains, raw fruits, raw vegetables) in my diet	24(2.61)	388(42.27)	396(43.14)	110(11.98)	2.64(±0.72)	10
14. Plan or select meals to include the “basic six” food groups each day	82(8.93)	523(56.97)	242(26.36)	71(7.73)	2.33(±0.74)	17
**Self-actualization**					2.81(±0.68)	
4. Work toward long-term goals in my life	30(3.27)	265(28.87)	397(43.25)	226(24.62)	2.89(±0.81)	5
5. Look forward to the future	37(4.03)	305(33.22)	387(42.16)	189(20.59)	2.79(±0.81)	7
13. Find each day interesting and challenging	65(7.08)	372(40.52)	317(34.53)	164(17.86)	2.63(±0.86)	11
24. Believe that my life has purpose	33(3.59)	271(29.52)	351(38.24)	263(28.65)	2.92(±0.85)	4
**Interpersonal Support**					2.87(±0.64)	
7. Maintain meaningful and fulfilling interpersonal relationships)	18(1.96)	228(24.84)	411(44.77)	261(28.43)	3(±0.78)	1
11. Spend time with close friends	43(4.68)	215(23.42)	405(44.12)	255(27.78)	2.95(±0.84)	2
17. Touch and am touched by people I care about	20(2.18)	262(28.54)	400(43.57)	236(25.71)	2.93(±0.79)	3
23. Find it easy to express concern, love and warmth to others.	92(10.02)	332(36.17)	335(36.49)	159(17.32)	2.61(±0.89)	13
**Health Responsibility**					2.11(±0.68)	
9. Discuss my health care concerns with qualified professionals	194(21.13)	472(51.42)	183(19.93)	69(7.52)	2.14(±0.83)	22
12. Have my blood pressure checked and know what it is	323(35.19)	416(45.32)	130(14.16)	49(5.34)	1.9(±0.83)	23
20. Seek information from health professionals about how to take good care of myself	208(22.66)	425(46.3)	207(22.55)	78(8.5)	2.17(±0.87)	20
21. Observe my body at least monthly for physical changes/danger sings	181(19.72)	424(46.19)	231(25.16)	82(8.93)	2.23(±0.87)	19
**Stress Management**					2.62(±0.63)	
15. Consciously relax muscles before sleep	101(11)	427(46.51)	252(27.45)	138(15.03)	2.47(±0.88)	15
18. Concentrate on pleasant thoughts at bedtime	59(6.43)	350(38.13)	327(35.62)	182(19.83)	2.69(±0.86)	9
19. Find constructive ways to express my feelings	56(6.1)	386(42.05)	356(38.78)	120(13.07)	2.59(±0.79)	14
22. Use specific methods to control my stress	35(3.81)	327(35.62)	394(42.92)	162(17.65)	2.74(±0.79)	8
**Total score**					60.76(±11.88)	

### Health status

Regarding self-reported health status, 9.37% (*n* = 86) and 5.77% (*n* = 53) of participants reported excellent and extremely poor health status, respectively. Approximately 20% of participants perceived poor and extremely poor health. The results of physiological health showed obesity in 19.93% (*n* = 183) by body mass index. Further, 29.30% (*n* = 269) and 8.93% (*n* = 82) of female and male participants, respectively, were obese as measured by body fat. Regarding psychological health, the results of Tung’s Depression Inventory showed that a minority of participants (11.22%, *n* = 103) experienced unstable emotions (Table 1).

### Correlation analysis

Health literacy only showed significant differences by frequency of exercise (*p* < 0.05) (S1); the higher the health literacy score, the higher the weekly frequency of exercise. Health-promoting lifestyle profile showed significant differences by gender, family financial status, and frequency of exercise (*p* < 0.05) (S2). The perceived health status and physical data did not show any statistically significant with participants’ characteristics. Only emotional status significant differences by place of residence and frequency of exercise (p < 0.05) by depression scale (S3). Health literacy and health-promoting lifestyle profile showed significant positive and negative correlation with perceived health status and depression, respectively (p < 0.05).

### Regression analysis

The regression analysis was designed to investigate which demographic factors related to the adolescents who participated in the study influenced health literacy, health-promoting lifestyle profile, and health status. All of discussion were based on descriptive data and final multivariate findings.

#### Health literacy

Health literacy only showed significant differences by weekly frequency of exercise.

#### Health-promoting lifestyle profile

The exercise frequency of ≥3 times weekly was a predictor of health literacy by linear regression analysis (*p* < 0.005). Variables predicted to affect health-promoting lifestyle profile included coming from a middle class family (*p* < 0.05) and exercise 1–2 times weekly (*p* < 0.001) and ≥ 3 times weekly (*p* < 0.0001) (Table 4).

**Table 4:** Factors of health-promoting lifestyle profile

	β	SE	T	***P***-value	R^**2**^
**Gender [reference = female (n = 726)]**					0.076
male (*n* = 192)	1.158	0.960	1.21	0.2283	
**Family income [reference = fair (n = 647)]**					
Above Well off (*n* = 217)	2.212	0.900	2.46	**0.0141**	
Poor (*n* = 54)	−0.671	1.626	−0.41	0.6801	
**Exercise Frequency [reference = 0 (*n*** **= 329**)**]**					
1 ~ 2 times (*n* = 412)	3.676	0.849	4.33	**< 0.0001**	
≥3 times (n = 177)	8.313	1.097	7.58	**< 0.0001**	

#### Health status

The participants who lived at home were 1.8 times more likely to experience emotional stability compared with those who live in dormitory or rented accommodation by logistic regression analysis (p < 0.05). Participants taking exercise 1–2 times/week were 1.7 times more likely to experience emotional stability than those who did not exercise (p < 0.05); furthermore, participants with exercise ≥3 times/week were 2.1 times more likely to experience emotional stability than those who did not exercise (p < 0.05; Table 5).

**Table 5:** Factors of emotional stability

	β	SE	Wald χ^**2**^	P-value	Odds Ratio (95% CI)
**Place of residence [reference = stayed in the school hostel or rented accommodation (n = 520)]**					
Stayed at home(*n* = 398)	0.593	0.224	6.981	**0.0082**	**1.809 (1.165–2.807)**
**Exercise Frequency [reference = 0 (n = 329)]**					
1 ~ 2 times (*n* = 412)	0.539	0.228	5.594	**0.018**	**1.714 (1.097–2.679)**
≥3 times (*n* = 177)	0.736	0.318	5.348	**0.0207**	**2.087 (1.119–3.892)**

*OR* odds ratio, *CI* confidence interval

## Discussion

The participants were from a junior college in central Taiwan, of which 80% were females and most participants were from the nursing department. These participants were freshmen aged 15–17 years, 80% were from central Taiwan. Approximately half (56.6%) of these students lived in a dormitory and most were living outside of their homes for the first time. Only 20% of participants exercised ≥3 times per week. The WHO (2010) recommends that children and adolescents aged 15–17 years should do moderate-to-vigorous exercise for at least 60 min daily. An exercise duration of > 60 min can provide additional health benefits. However, only 13.6% of students in Taiwan exercise for at least 60 min daily per week [29]. A global survey found that less than 20% of adolescents aged 13–15 years having moderate-to-vigorous exercise 60 min daily and do not exercise increases with age [30]. Adolescents who regularly participate in physical activities are likely to accept health behaviors and have better academic performance in school [29]. Cultivating the habit of physical activity seems an essential step of physical, psychological, cognitive, and social health benefits for adolescents. Therefore, it is crucial to strengthen the amount of physical activities in adolescents because this can develop better health behavior at a younger age.

### Health literacy

To our knowledge, this is the first study to explore adolescent health literacy and related factors in Taiwan. The results showed that approximately 30% of participants had insufficient or problematic health literacy. This findings is consist with results of other studies, approximately 40% of university students have insufficient or problematic health literacy in Taiwan [31]; 47% of participants had insufficient or problematic health literacy in European [12]. Loer et al., (2020) also found that 22.7% of adolescents in Germany had a low level of health knowledge [9]. Therefore, it can be deduced that there exists insufficient or problematic health literacy in different countries or age groups. However, the question was raised that HLS-EU-Q was developed for adults, and some items would be difficult for adolescents because of lack of experience in their lives [32]. Domanska et al., (2018) suggested either modifying the tool or seeking parents’ assistance for improving the validity of the measurement [32].

In this study, health information appraisal was the lowest aspect of health literacy. But Fleary et al., (2018) found inconsistent relationship between media health literacy and health information-seeking behaviors among adolescents based on the different health literacy instrument used [8]. Nutbeam (2000) mentioned that is critical and analytical health literacy is a higher level cognitive and social skills that applied in the critical analysis of information for better elucidation of events in life [33]. Most health information is transmitted by the Internet. If adolescents lack the ability to appraise and judge health information, this could affect health behavior and health [34]. Schools could adapt the concept of organizational health literacy to embed health literacy in the curriculum and make it easier for adolescents [35]. Thus, educating adolescents to recognize and detect health information needs to be addressed [9].

One of the lowest scores was for participants knowing which vaccinations they may need. Studies have shown that university students have negative attitudes towards and insufficient knowledge about vaccination [36, 37]. Since infectious/epidemic diseases have rapidly become common diseases in the twenty-first century, vaccines should not be overlooked. Adolescents are a vulnerable population considering that they are undergoing a difficult transition period in life [38]. It is essential to strengthen awareness of emerging epidemic diseases, such as coronavirus 2019, influenza, and acquired immunodeficiency syndrome, to protect the health of adolescents.

### Health-promoting lifestyle profile

The mean score for interpersonal support was the highest score in the health-promoting lifestyle profile. This result was consistent with results of other studies where interpersonal support scores were the highest [39, 40]. This factor could affects teenagers’ motivation for activity participation, which switches from parents and teachers to peers [41]. Thus, the influence of peers on health behavior in adolescents is considerably greater than from parents, teachers, or healthcare professionals [41]. Peer influence would be a key to improve the health-promoting lifestyle profile of adolescents.

Health responsibility had the lowest score in the health-promoting lifestyle profile, which echos the studies of Musavian (2014) in Iran and Tang et al. (2015) in Taiwan [20, 40]. Our study showed that the lowest scores were “measurement of own blood pressure and knowing one’s blood pressure” and “discussing personal health matters with professional medical staff” in the health responsibility dimension. This may be attributed to the younger age of adolescents, most of whom had never experienced a disease, and to the fact that only 19.28% of participants had self-perceived poor health but did not require special attention on health [40].

### Health status

We found that 19.93% of participants were obese. That 12.6% of participants who were obese was found in the 2017 health behavior survey by the Health Promotion Administration of the Ministry of Health and Welfare in Taiwan. The National Health and Nutrition Examination Survey in the United States showed that 20.6% of adolescents (aged 12–19 years) are obese and obesity rate has been steadily increasing during the 2015–2016 period [42]. Obesity is a global health issue, with the WHO listing obesity as one of the most important public health issues in the twenty-first century [43]. Simmonds (2016) found that the possibility of obese adolescents becoming obese adults is five times higher that of non-obese adolescents [44]. Obesity during adolescence will increase cardiovascular disease and diabetes risk in adulthood as well as cancer risk [45]. Therefore, adolescent obesity is an issue that should not be overlooked.

We found that 11.2% of adolescents experienced unstable emotions and depression. Studies have reported that anxiety and depression are common in adolescents and naturally occurs during adolescence [46]. Even at 18 years of age, more than 20% of adolescents may experience depressive episodes or anxiety [46]. Our study found that participants with high depression scores have lower health literacy and health-promoting lifestyle profile scores. The results are consistent with other studies where those with poorer lifestyle profile or low health literacy had higher rate of depression in junior high school [47]. Psychological health is highly related with health literacy in adolescents.

We found that adolescents who lived at home experienced emotional stability, whereas those who lived in dormitory or rented accommodation had poor emotional stability. Another study has reported that adolescents who lived outside of home often experienced stress and were unable to cope with stress [47]. The learning environment in the accommodation is vital to build interpersonal relationships, life values, and future life career. We recommend that school might need to pay more attention to students living outside of home to avoid maladjustment which may affect future health.

This study revealed that exercise significantly statistically differed with health literacy, health-promoting lifestyle profile, and depression. Participants who exercise more than three times per week have better health literacy and health-promoting lifestyle profile and greater emotional stability. This result echoes the findings that patients with poor health literacy exercised less and have poor health [15]. Others have suggested that regular exercise helps to decrease depressive tendencies in university students [48]. Cultivating interest in exercising and developing good exercise habits in adolescents is crucial for improving health literacy and health-promoting lifestyle profile and maintaining emotional stability. Physical education classes in tertiary educational institutions is the final opportunity for developing a regular exercise habit in their school life [49]. Identifying exercises that students prefer and mastering exercise techniques can assist in cultivating lifelong exercise habits [49]. We suggest that appropriate planning of physical education is significant for developing exercise habits and interest in adolescents.

### Limitations

First, the convenience sample was drawn from one school in the central district of Taiwan. Thus, the findings may not be generalized to all the adolescents in Taiwan. Second, the use of self-reported data may lead a reporting bias related to social desirability. Third, the HLS-EU-Q validity might be limited in an adolescent population and may need to be modified for better understanding adolescent health literacy. Furthermore, these data are cross sectional, longitudinal studies are needed to understand the modifiable factors as physical activity as potential target for better healthy lifestyle and health.

## Conclusions

We found that approximately 30% of adolescents have insufficient or problematic health literacy and have poor health information analysis and appraisal ability. We suggest that understanding and detecting health information needs to be taught. Health providers need pay more attention on adolescents’ knowledge about vaccination for better preventive health. Their health lifestyle profile was moderate and health responsibility was scored lowest. Adolescents should strengthen their ability to analyze and judge health information during their education and improve their health responsibilities. Health education should focus on using peer influence to improve the health-promoting lifestyle profile of adolescents.

Regarding health status, 19.93% of participants were obese and 11.2% of participants experienced poor emotional stability. A higher frequency of exercise corresponds to greater health literacy, better healthy lifestyle profile, and better psychological health status. Therefore, provision of suitable physical education training can allow adolescents to develop an exercise habit a crucial method for improving health literacy, developing a healthy lifestyle profile, and improving health.

## Supplementary Information

**Additional file 1: Supplement Table 1.** Correlation between participants’ characteristics and health literacy.

**Additional file 2: Supplement Table 2.** Correlation between participants’ characteristics and health promoting lifestyle profile (*n* = 918).

**Additional file 3: Supplement Table 3.** Correlation between Participants’ characteristics and depression scale.

## Data Availability

The datasets generated and/or analysed during the current study are not publicly available because of licensed but are available from the corresponding author on reasonable request. The data were used under license for this study and so are not publicly available.
