# Supplementary material for: Exploring the factors related to adolescent health literacy, health-promoting lifestyle profile, and health status
Source: BMC Public Health. 2021 Dec 1;21:2196. doi: 10.1186/s12889-021-12239-w (PMC8635084; doi:10.1186/s12889-021-12239-w)
Supplement: Supplementary file 1 — Additional file 1: Supplement Table 1. Correlation between participants’ characteristics and health literacy. [file 12889_2021_12239_MOESM1_ESM.docx]

Supplement Table 1. Correlation between participants’ characteristics and health literacy

| Variable | N | M (SD) | P | Scheffe  Post hoc |
| --- | --- | --- | --- | --- |
| Gender |  |  |  |  |
| Female | 726 | 36.13 (±6.07) | 0.899 |  |
| Male | 192 | 36.20 (±6.73) |  |  |
| Place of residence |  |  |  |  |
| Dormitory or rented  accommodation | 520 | 36.14 (±6.22) |  |  |
| Home | 398 | 36.16 (±6.20) | 0.966 |  |
| Ethnicity |  |  |  |  |
| Hokkien | 645 | 36.22 (±6.27) | 0.359 |  |
| Hakka | 204 | 36.17 (±6.18) |  |  |
| Province | 31 | 36.49 (±5.71) |  |  |
| Aboriginal | 38 | 34.40 (±5.55) |  |  |
| Family income |  |  |  |  |
| Above well off | 217 | 36.49 (±6.00) | 0.509 |  |
| Fair | 647 | 35.99 (±6.27) |  |  |
| Poor | 54 | 36.62 (±6.30) |  |  |
| Medical history |  |  |  |  |
| No | 893 | 36.15 (±6.23) | 0.905 |  |
| Yes | 25 | 36.00 (±5.63) |  |  |
| Smoking history |  |  |  |  |
| No | 900 | 36.13 (±6.20) | 0.585 |  |
| Yes | 18 | 36.94 (±6.84) |  |  |
| Alcohol consumption history |  |  |  |  |
| No | 899 | 36.12 (±6.17) | 0.352 |  |
| Yes | 19 | 37.46 (±7.92) |  |  |
| Exercise Frequency (per week) |  |  |  |  |
| 0 | 329 | 35.62 (±6.62) | 0.012 ^*^ | 3 > 1 |
| 1~2 times | 412 | 36.06 (±5.91) |  |  |
| ≥3 times | 177 | 37.32 (±5.98) |  |  |

^*^p < 0.05
