# Supplementary material for: Exploring the factors related to adolescent health literacy, health-promoting lifestyle profile, and health status
Source: BMC Public Health. 2021 Dec 1;21:2196. doi: 10.1186/s12889-021-12239-w (PMC8635084; doi:10.1186/s12889-021-12239-w)
Supplement: Supplementary file 2 — Additional file 2: Supplement Table 2. Correlation between participants’ characteristics and health promoting lifestyle profile (n = 918). [file 12889_2021_12239_MOESM2_ESM.docx]

Supplement Table 2. Correlation between participants’ characteristics and health promoting lifestyle profile (n=918)

| Variable | N | M (SD) | P | Scheffe  Post hoc |
| --- | --- | --- | --- | --- |
| Gender |  |  |  |  |
| Female | 726 | 60.20 (±11.33) | 0.014* |  |
| Male | 192 | 62.85 (±13.59) |  |  |
| Place of residence |  |  |  |  |
| Dormitory or rented  accommodation | 520 | 60.19 (±11.80) | 0.097 |  |
| Home | 398 | 61.50 (±11.96) |  |  |
| Ethnicity |  |  |  |  |
| Hokkien | 645 | 60.86 (±11.68) | 0.664 |  |
| Hakka | 204 | 60.99 (±12.02) |  |  |
| Province | 31 | 58.74 (±15.85) |  |  |
| Aboriginal | 38 | 59.32 (±11.03) |  |  |
| Family financial status |  |  |  |  |
| Above well off | 217 | 62.60 (±11.60) | 0.027* | 1 > 3 |
| Fair | 647 | 60.26 (±11.76) |  |  |
| Poor | 54 | 59.33 (±12.80) |  |  |
| Medical history |  |  |  |  |
| No | 893 | 60.75 (±11.85) | 0.985 |  |
| Yes | 25 | 60.80 (±13.33) |  |  |
| Smoking history |  |  |  |  |
| No | 900 | 60.75 (±11.90) | 0.797 |  |
| Yes | 18 | 61.06 (±11.15) |  |  |
| Alcohol consumption history |  |  |  |  |
| No | 899 | 60.69 (±11.83) | 0.229 |  |
| Yes | 19 | 64.00 (±14.09) |  |  |
| Exercise Frequency (per week) |  |  |  |  |
| 0 | 329 | 57.37 (±12.21) | <0.001 | 3 > 1, 2  2 > 1 |
| 1~2 times | 412 | 61.19 (±1.57) |  |  |
| ≥3 times | 177 | 66.03 (±2.12) |  |  |

*p < 0.05, **p < 0.01, ***p< 0.001
