# Supplementary material for: Exploring the factors related to adolescent health literacy, health-promoting lifestyle profile, and health status
Source: BMC Public Health. 2021 Dec 1;21:2196. doi: 10.1186/s12889-021-12239-w (PMC8635084; doi:10.1186/s12889-021-12239-w)
Supplement: Supplementary file 3 — Additional file 3: Supplement Table 3. Correlation between Participants’ characteristics and depression scale. [file 12889_2021_12239_MOESM3_ESM.docx]

Supplement Table 3. Correlation between Participants’ characteristics and depression scale

| Variable | Stable emotion  n (%) | Unstable emotion  n (%) | p |
| --- | --- | --- | --- |
| Gender |  |  |  |
| Female | 672 (92.56) | 54 (7.44) | 0.180 |
| Male | 183 (95.31) | 9 (4.69) |  |
| Place of residence |  |  |  |
| Dormitory or rented  accommodation | 475 (91.35) | 45 (8.65) | 0.014^*^ |
| Home | 380 (95.48) | 18 (4.52) |  |
| Ethnicity |  |  |  |
| Hokkien | 603 (93.49) | 42 (6.51) | 0.486 |
| Hakka | 190 (93.14) | 14 (6.86) |  |
| Province | 27 (87.10) | 4 (12.90) |  |
| Aboriginal | 35 (92.11) | 3 (7.89) |  |
| Family financial status |  |  |  |
| Above well off | 207 (95.39) | 10 (4.61) | 0.085 |
| Fair | 601(92.89) | 46 (7.11) |  |
| Poor | 47 (87.03) | 7 (12.96) |  |
| Medical history |  |  |  |
| No | 833 (93.28) | 60 (6.72) | 0.242 |
| Yes | 22 (88) | 3 (12) |  |
| Smoking history |  |  |  |
| No | 840 (93.33) | 60 (6.67) | 0.120 |
| Yes | 15 (83.33) | 3 (16.67) |  |
| Alcohol consumption history |  |  |  |
| No | 838 (93.21) | 61(6.79) | 0.379 |
| Yes | 17 (94.44) | 2 (5.56) |  |
| Exercise Frequency (per week) |  |  |  |
| 0 | 279 (84.80) | 50 (15.20) | 0.015^*^ |
| 1~2 times | 373 (90.53) | 39 (9.47) |  |
| ≥3 times | 163 (92.09) | 14 (7.91) |  |

^*^ p < 0.05
